# A141 DUPILUMAB IMPROVES CLINICAL, SYMPTOMATIC, ENDOSCOPIC, AND HISTOLOGIC ASPECTS OF EOE, REGARDLESS OF PRIOR SWALLOWED TOPICAL STEROID USE

**DOI:** 10.1093/jcag/gwac036.141

**Published:** 2023-03-07

**Authors:** A J Bredenoord, E S Dellon, A J Lucendo, M H Collins, A Khodzhayev, X Sun, K Patel, B Beazley, A Shabbir

**Affiliations:** 1 Amsterdam University Medical Center, Amsterdam, Netherlands; 2 University of North Carolina School of Medicine, Chapel Hill, NC, United States; 3 Hospital General de Tomelloso, Tomelloso, Spain; 4 Cincinnati Children’s Hospital Medical Center and University of Cincinnati College of Medicine, Cincinnati, OH; 5 Regeneron Pharmaceuticals, Inc., Tarrytown, NY; 6 Sanofi, Bridgewater, NJ, United States

## Abstract

**Background:**

Swallowed topical corticosteroids (STC) are a first-line treatment for eosinophilic esophagitis (EoE) but are not uniformly effective. Dupilumab (DPL), a fully human monoclonal antibody, blocks the shared receptor component for IL-4/IL-13, key and central drivers of type 2 inflammation. In Parts A and B of the phase 3 LIBERTY-EoE-TREET (NCT03633617) study, weekly DPL 300mg improved clinical, symptomatic, histologic, and endoscopic aspects of EoE and was generally well tolerated in adult and adolescent patients (pts) with EoE.

**Purpose:**

To assess the efficacy of weekly DPL 300mg vs placebo (PBO) at Week 24 in pts from Parts A and B with/without prior history of STC use, and from Part B with/without a history of inadequate response, intolerance, or contraindication to STCs.

**Method:**

Pts who received STCs for EoE within 8 weeks prior to baseline were excluded from the study. Co-primary endpoints at Week 24 were the proportion achieving peak eosinophil count (PEC) ≤6/high-power field (hpf) and the absolute change in Dysphagia Symptom Questionnaire (DSQ) score. Other secondary endpoints at Week 24 included: % change in PEC; absolute change in Histologic Scoring System (HSS) grade and stage scores and Endoscopic Reference Score (EREFS); % change in DSQ score.

**Result(s):**

At baseline, in Parts A and B combined, 84/122 (69%) and 87/118 (74%) of DPL- and PBO-treated pts had history of STC use. For pts treated with DPL vs PBO PEC≤6/hpf was achieved by 59.5% vs 3.4% of pts with, and 57.9% vs 12.9% without, prior STC use. Difference vs PBO (95% CI) in the absolute change in DSQ score was −13.27 (−18.03, −8.50) vs −5.21 (−12.41, 2.00) for pts with/without prior STC use. Difference vs PBO (95% CI) for pts with/without prior STC use were: % change in PEC −80.76 (−97.77, −63.75)/−84.87 (−112.16, −57.58); absolute change in EoE-HSS grade −0.77 (−0.87, −0.66)/−0.57 (−0.77, −0.38) and stage −0.77 (−0.87, −0.66)/−0.55 (−0.73, −0.36); absolute change in EREFS −3.86 (−4.70, −3.02)/−2.59 (−4.16, −1.02); % change in DSQ −34.5 (−47.75, −21.22)/-14.9 (−35.21, 5.36). DPL was generally well tolerated in the intent-to-treat population; the most common TEAEs for DPL/PBO were injection-site reactions (37.7/33.3%). In Part B, 38/80 (48%) and 39/79 (49%) of DPL- and PBO-treated pts had inadequate response/intolerance/contraindication to STCs. For DPL vs PBO PEC≤6/hpf was achieved by 55.3% vs 7.7% with, and 61.9% vs 5.0% of pts without, inadequate response/intolerance/contraindication to STC. Difference vs PBO (95% CI) for absolute change in DSQ score was −11.55 (−19.06, −4.04)/−7.08 (−13.75, −0.42) for pts with/without inadequate response/intolerance/contraindication to STCs.

**Conclusion(s):**

**Conclusion:** Regardless of prior STC use, in this pooled analysis from Part A and Part B of the EoE TREET Phase 3 Study, weekly DPL 300mg demonstrated substantial improvements in clinical, histologic, and endoscopic study endpoints at Week 24 in adults and adolescents with EoE.

**Please acknowledge all funding agencies by checking the applicable boxes below:**

Other

**Please indicate your source of funding;:**

Research sponsored by Sanofi and Regeneron Pharmaceuticals, Inc.

**Disclosure of Interest:**

A. Bredenoord Shareholder of: SST, Grant / Research support from: Bayer, Nutricia, SST, Consultant of: Arena Pharmaceuticals, AstraZeneca, Calypso Biotech, Dr Falk, EsoCap, Gossamer Bio, Laborie, Medtronic, RB Pharma, Regeneron Pharmaceuticals, Inc., Robarts Clinical Trials, E. Dellon Grant / Research support from: Research funding; Adare Pharma Solutions, Allakos, GSK, Meritage Pharma, Miraca Life Sciences, Nutricia, Receptos/BMS, Regeneron Pharmaceuticals, Inc., Shire. Educational grant; Allakos, Banner Pharmaceuticals, Holoclara, Consultant of: Abbott, Adare Pharma Solutions, Aimmune Therapeutics, Alivio Therapeutics, Allakos, Arena Pharmaceuticals, AstraZeneca, Banner Pharmaceuticals, Biorasi, Calypso Biotech, Enumeral, EsoCap, Gossamer Bio, GSK, Receptos/BMS, Regeneron Pharmaceuticals, Inc., Robarts Clinical Trials, Salix Pharmaceuticals, Shire/Takeda, A. Lucendo Grant / Research support from: Dr Falk, Regeneron Pharmaceuticals, Inc., Consultant of: Dr Falk, EsoCap, M. Collins Grant / Research support from: Receptos/BMS, Regeneron Pharmaceuticals, Inc., Shire, Consultant of: Allakos, AstraZeneca, BMS, EsoCap, Regeneron Pharmaceuticals, Inc., Shire, A. Khodzhayev Shareholder of: Regeneron Pharmaceuticals, Inc., Employee of: Regeneron Pharmaceuticals, Inc., X. Sun Shareholder of: Regeneron Pharmaceuticals, Inc., Employee of: Regeneron Pharmaceuticals, Inc., K. Patel Employee of: Sanofi, B. Beazley Shareholder of: Regeneron Pharmaceuticals, Inc., Employee of: Regeneron Pharmaceuticals, Inc., A. Shabbir Shareholder of: Regeneron Pharmaceuticals, Inc., Employee of: Regeneron Pharmaceuticals, Inc.

